# LDH and NLR, as inflammatory markers, the independent risk factors for COVID-19 complicated with respiratory failure in elderly patients

**DOI:** 10.12669/pjms.40.9.8728

**Published:** 2024-10

**Authors:** Shan Wang, Jia Liu, Song Hu, Yongjun Mao

**Affiliations:** 1 Shan Wang, Department of Geriatric Medicine, The Affiliated Hospital of Qingdao University, Qingdao, Shandong 266003, China; 2 Jia Liu, Department of Geriatric Medicine, The Affiliated Hospital of Qingdao University, Qingdao, Shandong 266003, China; 3 Song Hu, Department of Geriatric Medicine, The Affiliated Hospital of Qingdao University, Qingdao, Shandong 266003, China; 4 Yongjun Mao, Department of Geriatric Medicine, The Affiliated Hospital of Qingdao University, Qingdao, Shandong 266003, China

**Keywords:** COVID-19, LDH, Senior, Respiratory failure

## Abstract

**Objective:**

Lactate dehydrogenase (LDH) is an enzyme that is responsible for the production of lactic acid, which is a necessary byproduct when the body does not have enough oxygen. LDH levels in the blood can be used as a marker to predict mortality in patients with ARDS, severe COVID-19, and cancer. To analyze the clinical characteristics of COVID-19 in the elderly and the correlation between LDH and respiratory failure in COVID-19 patients, to improve the identification and management of this type of pneumonia by clinicians.

**Methods:**

This was a single-center retrospective study. We performed routine laboratory tests in 105 COVID-19 patients admitted to the affiliated hospital of Qingdao University (Qingdao, China) from October 1, 2022 to February 1, 2023. The diagnosis of respiratory failure was established based on the results of blood gas analysis upon admission.

**Results:**

The median age was 79 years. Among all univariable parameters, LDH, neutrophil to lymphocyte ratio (NLR) and Prothrombin Time (PT) were significantly independent risk factors of RF in elderly COVID-19 patients. LDH (AUC=0.829) also had a maximum specificity (96.5%), with the cutoff value of 280.5.

**Conclusion:**

The levels of LDH, NLR, and PT may serve as potential indicators for elderly COVID-19 patients combined with respiratory failure. LDH, NLR and PT assays can be beneficial for patients who need closer respiratory monitoring and more aggressive supportive care to prevent a negative prognosis.

## INTRODUCTION

The primary symptoms of COVID-19 infection are typically a dry cough and fever, which can potentially develop into interstitial pneumonia and ultimately lead to respiratory failure.^1^ Previous clinical experience has revealed that some elderly patients may not exhibit symptoms of dyspnea, despite having reduced peripheral blood oxygen saturation and severe lung involvement, as confirmed by lung CT scans.^2^-^4^

Respiratory failure is a condition in which the respiratory system fails in oxygenation or carbon dioxide elimination or both. In hypoxemic respiratory failure, ventilation-perfusion (V = Q) mismatch results in the decrease of PaO2 to below 60 mm Hg with normal or low PaCO2. In hypercapnic respiratory failure, V = Q mismatch results in the increase of PaCO2 to above 50 mm Hg.^5^,^6^ Clinicians are tasked with the challenge of identifying which COVID-19 patients are at a high risk of experiencing respiratory failure. Risk factors for respiratory failure in patients with COVID-19 include old age, comorbidities such as hypertension and diabetes.^1^,^7^,^8^ Almost half of the population infected by COVID-19 has underlying problems mainly diabetes, cerebrovascular and cardiovascular problems. The older people with the weak or compromised immune system are more susceptible to this disease.^9^ Nevertheless, our understanding of the pathogenesis of COVID-19 remains incomplete. The central role in disease progression and severity is attributed to the inflammatory cytokine storm and viral evasion of the cellular immune response.^10^,^11^ In a meta-analysis by Henry et al., severe and fatal COVID-19 patients had significantly elevated inflammation, heart and muscle damage, liver and kidney function, and coagulation markers, particularly IL-6, IL-10, and serum ferritin, which is a strong differential indicator of severe disease.^2^,^12^

LDH is an enzyme found in most cells in the body that helps convert pyruvate to lactate and vice versa, while also converting NADH to NAD^+^.^13^-^16^ Although traditionally used as a marker of heart damage, abnormal LDH levels may also indicate multiorgan injury and decreased oxygen supply due to increased glycolysis.^14^ Additionally, high levels of lactate due to infection or tissue damage can trigger activation of metalloproteinases and promote angiogenesis mediated by macrophages.^17^ Severe COVID-19 infection can lead to tissue damage and the release of LDH, present in lung tissue. This can result in higher levels of LDH in the circulation, as severe interstitial pneumonia is a common feature of the disease. 17,18 Studies have shown that elevated LDH levels in hospitalized patients predict poor prognosis.^19^,^20^ The current predictors and treatments for COVID-19 are based on experience with previous coronavirus outbreaks, such as SARS, and other viral respiratory infections. Elevated LDH levels may indicate a more significant role of multiorgan damage and failure in COVID-19 pathology, which can affect clinical outcomes in patients.^21^

This study proves that certain laboratory markers commonly used to detect tissue damage and inflammation can predict the likelihood of respiratory failure in COVID-19 patients. This is particularly useful in community hospitals where CT scans may not be readily available.

## METHODS

A retrospective observational study was conducted from October 1, 2022 to February 1, 2023, including 105 elderly patients at the Affiliated Hospital of Qingdao University who confirmed COVID-19. The positive result of real-time reverse transcription polymerase chain reaction (RT-PCR) of nasopharyngeal swab samples were used as diagnosis of COVID-19.

We collected laboratory data from demographic, epidemiological, clinical, physical examination findings, and electronic medical records. Laboratory assessments included complete blood count, liver and kidney function, markers of cardiac injury, electrolytes and C-reactive protein and other parameters. According to the results of blood gas analysis and the diagnostic criteria of respiratory failure, we divided the COVID-19 patients into respiratory failure group and non-respiratory failure group. Patient information was confidentially protected and this study was approved by the Ethics Committee of the Affiliated Hospital of Qingdao University (QYFY WZLL 27977).

### Statistical analysis:

Quantitative variables were expressed as means ± standard deviation, t-test was used for significance. Nonparametric variables were expressed as medians and interquartile ranges, or simply ranges, and differences were compared using the Mann-Whitney U test or the Kruskal-Wallis test. Continuous and categorical variables were summarized as counts and percentages and tested for significance by chi square test or Fisher’s exact test. Univariate and multivariate logistic regression models were used to examine risk factors associated with COVID-19 adverse outcome. The sensitivity and specificity of these risk factors in patient diagnosis were represented and analyzed by receiver operating characteristic curve (ROC curve). P value (two-trailed) <0.05 was considered significant. All analysis were performed by asps 26.0 software (IBM SPSS, USA).

## RESULTS

### Demographics and clinical characteristics of COVID-19 patients:

The diagnosis of COVID-19 was made based on guidance from the World Health Organization. A total of 105 elderly patients were included in this study, with 37 respiratory failure (RF) and 68 non-RF cases (Table-I), 71 cases (67.6%) were male, of which 75.7% were RF (p=0.193). The median age was 79 (71-85) years. There was no significant difference in underlying conditions between the two groups (p >0.05).

**Table-I:** Demographic and clinical characteristics of COVID-19 patients.

	Total (N=105)	Non-RF patients (N=68)	RF patients (N=37)	P value
Age (Years)	79.00 (71.00,85.00)	79.50 (70.25,85.00)	79.00 (71.50,85.00)	0.875
Gender (Female)	-	-	-	0.193
Male	71(67.6%)	43(63.2%)	28(75.7%)	
Female	34(32.4%)	25(36.8%)	9(24.3%)	
History	-	-	-	-
Hypertension	61(58.1%)	42(61.8%)	19(51.4%)	0.302
CHD	34(32.4%)	20(29.4%)	14(37.8%)	0.378
MI	8(7.6%)	4(5.9%)	4(10.8%)	0.600
HF	4(3.8%)	3(4.4%)	1(2.7%)	1.000
COPD	12(11.4%)	6(8.8%)	6(16.2%)	0.414
DM	44(41.9%)	28(41.2%)	16(43.2%)	0.838
Tumor	11(10.5%)	5(7.4%)	6(16.2%)	0.279
Smoke	12(11.4%)	5(7.4%)	7(18.9%)	0.145
Drink	8(7.6%)	4(5.9%)	4(10.8%)	0.600
BMI (kg/m^2^)	24.79±4.31	24.74±4.75	24.90±3.25	0.855

CHD: Coronary heart disease; MI: Myocardial infarction; HF: Heart failure; COPD: Chronic obstructive pulmonary disease; DM: Diabetes mellitus.

### Laboratory indices of COVID-19 patients:

Compared to the non-RF patients, White blood cell (p=0.001), Neutrophil (p<0.001), Lymphocyte (p<0.05), NLR (p<0.001), CD4 (p=0.003), IL-6 (p=0.005), CRP (p<0.001), PCT (p<0.01), AST (p=0.004), PAB (p<0.001), LDH (p<0.001), Urea (p=0.026), cTn-I (p=0.025), D-dimer (p<0.001) and PT (p<0.001) in RF patients were significantly higher at admission (Table-II).

**Table-II:** Laboratory indices of COVID-19 patients.

	Total (N=105)	Non-RF patients (N=68)	RF patients (N=37)	P value
White blood cell (10^9/L)	6.63(4.63, 8.42)	6.18(4.49, 7.56)	8.20(5.53, 10.34)	0.001***
Neutrophil (10^9/L)	5.17(3.23, 6.66)	4.35(2.58, 6.00)	6.49(4.36, 14.51)	<0.001***
Lymphocyte (10^9/L)	0.95(0.60, 1.34)	1.04(0.73, 1.39)	0.66(0.42, 1.26)	0.015**
NLR	5.15(2.95, 10.60)	4.03(2.35, 7.68)	10.38(4.21, 14.76)	<0.001***
CD4 (cells/uL)	209.00(146.50, 352.00)	351.00(233.00, 589.00)	172.50(137.75, 206.00)	0.003**
IL-6 (pg/mL)	16.78(4.65,43.07)	10.81(3.38,25.84)	28.50(9.94,82.29)	0.005**
CRP (mg/L)	26.86(6.94,58.55)	17.74(5.55,33.38)	59.02(25.95,101.47)	<0.001***
PCT (ng/mL)	0.08(0.05,0.21)	0.06(0.04,0.10)	0.17(0.09,0.28)	<0.001***
ALT (U/L)	19.70(14.00,34.00)	18.50(12.00,30.75)	20.00(16.50,44.65)	0.081
AST (U/L)	25.00(18.00,35.85)	22.50(17.25,29.00)	29.00(21.50,68.30)	0.004**
ALB (g/L)	33.65(31.30,36.25)	33.50(31.50,36.30)	33.80(28.65,35.35)	0.316
PAB (mg/L)	118.45(82.45,184.50)	145.20(100.00,193.20)	82.90(48.70,124.05)	<0.001***
PNI	38.23(34.48,41.90)	38.30(35.30,42.20)	37.35(32.25,41.45)	0.069
LDH (U/L)	221.00(177.00,284.50)	204.00(172.00,243.50)	327.00(247.50,633.80)	<0.001***
Urea (mmol/L)	6.18(4.75,8.23)	5.93(4.62,8.02)	7.17(5.15,10.04)	0.026*
Crea (mmol/L)	87.00(70.21,104.00)	88.30(70.80,99.75)	87.00(68.50,116.05)	0.609
cTn-I (ng/mL)	0.007(0.001,0.031)	0.005(0.001,0.016)	0.013(0.002,0.123)	0.025*
D-dimer (ng/mL)	460.00(330.00,795.00)	420.00(300.00,570.00)	785.00(460.00,3087.50)	<0.001***
PT (sec)	10.90(1.02,13.63)	1.13(0.98,12.78)	13.30(10.45,14.58)	<0.001***

NLR: neutrophil to lymphocyte ratio; PAB: Prealbumin; PNI: prognostic nutritional index.

*: P≤0.05;

** P≤0.01;

*** P≤0.001.

### Independent risk factors of adverse outcome of COVID-19 patients:

To assess the risk factors of the demographics and laboratory indicators on the COVID-19 patients combined with respiratory failure, logistic regression analysis was performed on the parameters of significant difference. In univariable analysis, we found that white blood cell, NLR, IL-6, CRP, LDH, D-dimer, PT level were all associated with COVID-19 elderly patients combined with respiratory failure. Meanwhile, CD4^+^ cells and PAB were protective factors (OR<1) for COVID-19 elderly patients. Then we included age, gender, white blood cell, NLR, IL-6, CRP, LDH, D-dimer, PT level for a multivariate regression analysis, as a result, NLR (OR=2.112, 95%CI:1.053, 4.234, p=0.035), LDH (OR=1.031, 95%CI: 1.003, 1.061, p=0.030), PT (OR=2.008, 95%CI: 1.048, 3.848, p=0.036) were found to be independent risk factors for the COVID-19 elderly patients combined with respiratory failure. (Table-III)

**Table-III:** Univariate and multivariate logistic regression analysis of COVID-19 patients combined with respiratory failure.

	Univariable OR(95% CI)	P value	Multivariable OR(95% CI)	P value
Age (years)	1.005(0.964, 1.048)	0.821	0.873 (0.732, 1.042)	0.133
Gender(female)	1.809(0.737, 4.442)	0.196	0.001 (0.000, 1.163)	0.055
White blood cell(10^9/L)	1.268(1.083, 1.483)	0.003	0.751 (0.408, 1.380)	0.356
Neutrophil(10^9/L)	1.052(0.971, 1.139)	0.214		
Lymphocyte(10^9/L)	0.438(0.184, 1.044)	0.062		
NLR	1.113(1.038, 1.194)	0.003	2.112 (1.053, 4.234)	0.035*
CD4^+^(cells/uL)	0.985(0.973, 0.998)	0.023		
IL-6(pg/mL)	1.019(1.002, 1.036)	0.025	1.030 (0.977, 1.085)	0.272
CRP(mg/L)	1.033(1.017, 1.049)	<0.001	0.996 (0.961, 1.031)	0.802
PCT(ng/mL)	1.082(0.931, 1.259)	0.305		
PAB(mg/L)	0.989(0.982, 0.996)	0.002	1.018 (0.990, 1.047)	0.207
LDH(U/L)	1.022(1.012, 1.032)	<0.001	1.031 (1.003, 1.061)	0.030*
Urea(mmol/L)	1.122(0.997, 1.262)	0.056		
cTn-I(ng/mL)	7.677(0.382, 154.209)	0.183		
D-dimer(ng/mL)	1.000(1.000, 1.001)	0.021	1.000 (0.999, 1.001)	0.613
PT(sec)	1.130(1.053, 1.213)	0.001	2.008 (1.048, 3.848)	0.036*

*: P≤0.05.

### The predictive factors for identification of COVID-19 elderly patients combined with respiratory failure:

**Fig.1:**
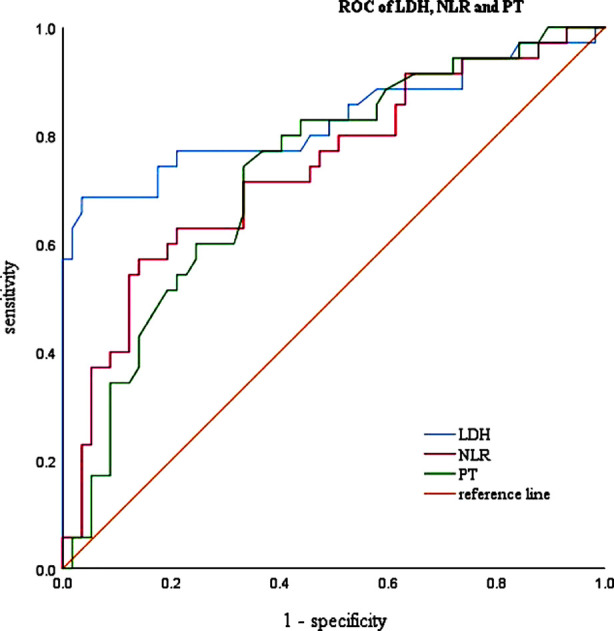
Receiver operating characteristic (ROC) curves for LDH, NLR and PT in patients with respiratory failure.

The diagnostic value of these selected parameters were evaluated by receiver operating characteristic (ROC) curve and area under ROC curve (AUC). As showed in Fig.1, the area under curve(AUC=0.829) implied a perfect accuracy of the serum LDH level more than 280.50 U/L in COVID-19 elderly patients as a predictive factor for identification of respiratory failure, with the high specificity (96.5%) and sensitivity (68.6%). Besides, the NLR over 9.35 and PT over 10.95 (s) showed relative moderate accuracy with AUC=0.737 and AUC=0.730 (Fig.1).

## DISCUSSION

In this study, we analyzed the clinical features in 105 patients with COVID-19. COVID-19 is one of the important causes of increased hospitalization and mortality in elderly patients. The reason for this phenomenon is that with the increase of age, the function of mucociliary clearance disorder and alveolar defense function in the elderly are damaged to varying degrees, and often accompanied by other chronic diseases such as cerebrovascular disease, resulting in swallowing dysfunction and ineffective cough, making infection more likely to occur^22^ At present, COVID-19 in the elderly has caused serious economic and social burden, so it is important to identify the severity of the disease as early as possible and select the appropriate treatment.^23^

Among the risk factors we examined in this study, we found that LDH had positive relationship with RF. LDH is a major player in glucose metabolism, which is present in systemic tissues and catalyzes the production of lactate from pyruvate. It is released when the plasma membrane of the cell is damaged.^24^ Previous studies have also established the importance of LDH as an indicator of lung disease. In a study of Epstein-Barr virus (EBV), researchers found that EBV infected B cells had more LDH transcripts than uninfected B cells.^25^ In addition, LDH levels are elevated in the serum of patients with Pneumocystis pneumoniae and may be associated with lung injury.^26^

Among patients infected during the 2009 influenza A (H1N1) pandemic, 77.8% of laboratory tests showed LDH>225 U/L involving the lung, and there was no significant difference between adults and children, suggesting that elevated LDH is associated with various pathogens such as the virus and with lung injury.^27^,^28^ In addition, a case of significantly elevated LDH in a patient with human Zika virus infection was reported in 2017, which was associated with 70% mortality in a further animal study of Zika virus infection.^11^,^29^ They considered LDH as an indicator of multi-organ injury rather than just affecting liver or cardiac function. Our study showed that LDH (OR=1.031, 95% CI: 1.003-1.061, p=0.030) was an independent risk factor predicting COVID-19. Then, we analyzed the diagnostic value of the LDH. ROC curves displayed that LDH had a good diagnostic efficiency (AUC=0.829).

NLR is a systemic inflammatory marker that has been widely used in many situations, such as predicting the prognosis of cardiovascular disease in patients with sepsis who die in the hospital.^30^ NLR has been proposed as a novel predictor of mortality in various diseases, such as heart failure and several cancers;^31^ the neutrophil-to-lymphocyte ratio was found to be significantly higher in COVID-19 patients than in healthy controls.^32^ High levels of NLR in serum indicate an unbalanced inflammatory response, which is caused by increased neutrophils and decreased lymphocyte counts.^33^ Numerous studies all over the world have revealed that the destruction of lymphocyte and other immune cells by coronavirus leading to the weakening of cellular immune system.^9^,^34^ Simultaneously, study have found that SARS-CoV-2 infection and the inflammatory storm it brings can lead to the dysfunction of the body’s coagulation/fibrinolysis system through a variety of mechanisms, and increase the risk of thromboembolism. In our study, we found that NLR and PT were significantly higher in RF patients than in non-RF patients, which was consistent with the findings of a previous study.^35^

### Limitations:

The limitations of this study are that the study was a single-center retrospective study and the sample size was small.

## CONCLUSION

In conclusion, elevated LDH, NLR and PT values were associated with an increased risk of adverse outcome of COVID-19 disease. More importantly, elevated LDH was associated with a 1.031-fold increase in mortality. Therefore, patients’ LDH levels should be closely monitored for signs of disease progression or decompensation. Since the LDH levels used in the study were measured at admission or at the earliest time during hospitalization, LDH levels at admission could be included in future risk stratification models for COVID-19 severity and mortality. Larger studies are needed to confirm these findings. These studies offer guidance for potential future outbreaks.

### Author’s Contributions:

**SW and JL:** Preparation of the material and data collection.

**SW and SH:** Data analysis.

**SW and YM:** Prepared the manuscript

**YM:** Responsible for the accuracy of the study.

All authors read and approved the final manuscript and contributed to the conception and design of the study.
